# Evidence from Characteristics and Comorbidities Suggesting That Asperger Syndrome Is a Subtype of Autism Spectrum Disorder

**DOI:** 10.3390/genes13020274

**Published:** 2022-01-30

**Authors:** Stephen M. Edelson

**Affiliations:** Autism Research Institute, San Diego, CA 92116, USA; director@autism.org; Tel.: +1-619-281-7165

**Keywords:** Asperger syndrome, autism spectrum disorder, autistic disorder, subtyping, DSV-V

## Abstract

The current version of the American Psychiatric Association’s Diagnostic and Statistical Manual of Mental Disorders (DSM-V) does not consider Asperger syndrome a diagnostic category. This study was undertaken to see if there is evidence that this diagnosis should be reinstated. An online survey was conducted to examine symptoms and behaviors associated with the current diagnostic criteria of autism spectrum disorders (ASD) (DSM-V), and those associated with Asperger syndrome based on the previous version (DSM-IV-TR). The study also examined other characteristics historically associated with autism, as well as impairments often reported in infancy/young childhood and medical comorbidities frequently associated with autism. The sample included 251 individuals who had received a diagnosis of Asperger syndrome and 1888 who were diagnosed with autism or ASD. Numerous similarities and differences were found between the two groups. The findings are discussed in relation to reestablishing Asperger syndrome as a valid diagnostic category as well as a subtype of ASD.

## 1. Introduction

From 1994 to 2012, the American Psychiatric Association recognized autistic disorder and Asperger syndrome [1,2]. The criteria for autistic disorder included onset prior to age three years; impairments in communication and social interaction; and restricted, repetitive, and stereotyped patterns of behavior, interests, and activities. The criteria for Asperger syndrome were similar to autistic disorder except for a minimum age of onset in addition to minimal delays in cognition, adaptive skills, and language.

In 2013, these diagnoses were combined into a general category termed “autism spectrum disorder” [3]. The criteria were similar to the “autistic disorder” diagnostic category defined in the previous version of the DSM. The new version emphasized impairments in social communication rather than viewing challenges in communication as a trait independent of social interaction, and included hyper-reactivity, hypo-reactivity, or unusual interests with regard to processing sensory information.

One reason for redefining autism was to clarify the core symptoms and behaviors associated with autism. It was argued that a redefinition of autism would lead to more accurate diagnosing of ASD [4]. Another reason for redefining autism was to reduce the number of those individuals diagnosed with ASD who exhibited relatively mild symptoms and behaviors. David J. Kupler, the chairperson of the APA’s autism task force, was quoted in the *New York Times* as stating, “We have to make sure not everybody who is a little odd gets a diagnosis of autism or Asperger Disorder. It involves a use of treatment resources. It becomes a cost issue” [5]. 

Currently, individuals who would have met the criteria for Asperger syndrome using the DSM-IV-TR but do not have severe symptoms are no longer considered as having a form of autism. Consequently, they no longer qualify for needed government-funded services as well as insurance coverage for treatment [6,7,8]. 

It is too early to evaluate the effects of removing the diagnosis of Asperger syndrome from the DSM-V. Initial research indicates that between 50% and 75% of those qualified for a diagnosis of Asperger syndrome based on the DSM-IV-TR criteria also met the requirements for a diagnosis of ASD based on the DSM-V criteria [9,10,11]. The concern is whether those who no longer qualify for an ASD diagnosis, 25% to 50%, are currently experiencing a decrease in their quality of life or will experience a decreased quality of life in the future. Much more research will likely be published on this issue prior to the next edition of the DSM. 

On a different front, proponents of reinstating the Asperger syndrome diagnosis argue that this will lead researchers to take an interest in studying individuals with this particular diagnosis rather than overlooking them because they do not fall within “acceptable” diagnostic criteria. In addition, they argue that identifying any subgroup of autistic individuals with distinguishable characteristics is likely to lead to improvements in diagnosis and to more effective interventions leading to a better prognosis. 

Mounting evidence suggests that there are a number of unique subtypes of autism that share common symptoms and behaviors [12,13]. Furthermore, researchers have already identified numerous biomarkers specific to Asperger syndrome [14]. Examples include genetics [15,16], metabolites [17], stem cells [18], and neurostructures [19,20]. 

Researchers have also explored subtyping by comparing Asperger syndrome to “high functioning autism” (HFA) [21,22]. For example, de Giambattista and colleagues considered individuals as HFA who had an intellectual quotient (IQ) of average or above in addition to a diagnosis of either autistic disorder or Pervasive Developmental Disorder-Not Otherwise Specified (DSM-IV-TR) [23]. Comparing these individuals to those diagnosed with Asperger syndrome, the researchers identified differences in cognition, language, school functioning, and comorbidities, and suggested that “an Asperger syndrome empirical distinction within autism spectrum disorder should be clinically useful.” 

Since research on biomarkers is still ongoing, the argument over re-establishing Asperger syndrome as a formal diagnosis currently needs to focus on and expand upon studies such as that of de Giambattista et al. by examining the characteristics of individuals with an Asperger diagnosis that are similar to the characteristics of those diagnosed with autism spectrum disorder, as well as differences that set them apart [23]. Thus, the current research, using the results from a relatively large survey, compared individuals diagnosed with Asperger syndrome to those diagnosed with autism and ASD (autism/ASD). Comparisons were made with respect to early signs of impairment, other commonly reported symptoms and behaviors, and medical comorbidities. In contrast to previous research, which typically has explored individual issues (for instance, medical comorbidities), this study evaluated a wide range of similarities and differences within the study groups.

## 2. Materials and Methods

Two questionnaires were employed in this study. The first was the Diagnostic Checklist Form E-2, a questionnaire written by researcher and parent advocate Bernard Rimland [24]. The E-2 has been administered throughout the autism community for over 50 years, and research has supported its validity, although it is not clear whether it can differentiate degrees of autism [25,26]. The second, more recent questionnaire used in this study was designed to identify a variety of medical comorbidities. Two publications recently reported significant findings using this checklist [27,28].

Two of the first questions asked were whether the individual had received a diagnosis and what the diagnosis was. Respondents included 251 individuals with Asperger syndrome and 1888 individuals with autism/ASD, for a total of 2139 cases. Of those diagnosed with autism/ASD, 83.9% were males and 16.1% were females. Of those diagnosed with Asperger syndrome, 81.7% were males and 18.3% were females. 

Age was classified into five categories on the E-2 checklist. In the autism/ASD group, 2.4% percent of individuals were under three years of age; 7.8% were between three and four years of age; 7.6% were between four and five years of age; 10.7% were between five and six years of age; and 71.5% were over six years of age. All individuals with Asperger syndrome were over the age of six years. The exact age of diagnosis was not asked in the survey.

The data for the questionnaires were obtained between 2015 and 2017 using a professional online survey service (www.Alchemer.com (accessed on 31 December 2017)). The research project was announced on the Autism Research Institute’s website (autism.com (accessed on 23 July 2021)) and in the Institute’s monthly e-newsletters.

The questionnaires were completed by mothers (86.6%), fathers (10.3%), other relatives including aunts, grandparents, siblings, and step-parents (2.5%), professionals including psychologists and special education teachers (0.003%), and individuals with ASD (0.008%). The high number of mothers who completed the questionnaires is consistent with research indicating that they are more involved in raising their children than the fathers [29,30]. 

Tallies were calculated using an asp.net program that queried a Microsoft Access database. A non-parametric statistical test, chi-square, was employed since the data were categorical. An online program, Social Science Statistics (www.socscistatistics.com (accessed on 23 July 2021)) calculated the chi-square values and probability levels. The α level was set at *p* < *0*.05. A Bonferroni Correction for multiple comparisons was calculated into the analyses. The α level was *p* = 0.0003.

Not every question on the two checklists was answered. With respect to those questions under examination, the percentage of missing data ranged from 0.0009% (i.e., “Present age of child”) to 2.0% (i.e., “Has your child had a seizure?” and “Does the child like to spin things like jar lids, coins, or coasters?”).

Due to the relatively large number of questions (170 in all), symptoms and behaviors were selected a priori for analysis if they were consistent with the criteria for Asperger syndrome and autistic disorder in the DSM-IV-TR, and the criteria for autism spectrum disorder in the DSM-V. In addition, commonly reported early signs of autism [24,31] and medical comorbidities [32] were examined. Responses such as “don’t know” or “not sure” were not included in the analyses. 

## 3. Results

The results are presented with respect to non-significant and significant findings. The former reflect similarities between Asperger syndrome and autism/ASD, whereas the latter reflect the uniqueness of each group.

### 3.1. Genetic Features

There were no differences between those with Asperger syndrome and autism/ASD with regard to sex ratio, χ^2^(1, *n* = 2130) = 0.78, *p* = 0.38. Those with Asperger syndrome were more likely to have a first degree relative with autism on the mother’s and/or father’s side of the family, χ^2^(1, *n* = 2139) = 21.95, *p* = 0.000003, and χ^2^(1, *n* = 2139) = 25.35, *p* = 0.0000005, respectively. There was no statistical difference between the two groups with respect to having a blood relative with schizophrenia, χ^2^(1, *n* = 2139) = 0.65, *p* = *0*.42, and/or depression, χ^2^(1, *n* = 2139) = 12.16, *p* = 0.005. See Table 1.

### 3.2. Early Signs (Age Two Years and under)

Regarding several of the early signs of autism, there were no differences between individuals with Asperger syndrome and those with autism/ASD. This included spinning objects, χ^2^(1, *n* = 2098) = 2.74, *p* = *0*.10, lining up things, χ^2^(1, *n* = 1966) = 3.63, *p* = *0*.06, rhythmic or rocking activity, χ^2^(2, *n* = 2114) = 2.55, *p* = 0.28, rocking during infancy, χ^2^(2, *n* = 1859) = 3.36, *p* = 0.19, difficulties being held, χ^2^(3, *n* = 2036) = 7.80, *p* = 0.05, avoiding people, χ^2^(1, *n* = 2117) = 0.13, *p* = 0.72, repeating words (i.e., echolalia), χ^2^(1, *n* = 2102) = 2.17, *p* = 0.14. and perceiving certain sounds as painful, χ^2^(1, *n* = 2117) = 3.19, *p* = 0.07. See Table 2.

### 3.3. Commonly Reported Descriptors

In contrast to the findings reported above, there were numerous differences between those diagnosed with Asperger syndrome and autism/ASD involving motor impairment and sensory sensitivities. Regarding the former, those with Asperger syndrome were more likely to have physical coordination challenges whereas those with autism/ASD were more likely to be considered graceful, χ^2^(2, *n* = 2103) = 49.74, *p* = 0.00000000002. Furthermore, those with autism/ASD were more likely to hold their hands in strange postures, χ^2^(1, *n* = 2116) = 20.72, *p* = 0.000005. 

Regarding sensory sensitivities, those with Asperger syndrome were more likely as infants to react to sensory stimuli (e.g., bright lights and colors, unusual sounds), whereas those with autism/ASD were less responsive, χ^2^(2, *n* = 2126) = 38.97, *p* = 0.000000003. In addition, those with Asperger syndrome were more likely to suffer from tactile sensitivities, such as disliked being touched or held, χ^2^(1, *n* = 2109) = 27.28, *p* = 0.0000002, and resistant to new articles of clothing, χ^2^(1, *n* = 2117) = 15.44, *p* = 0.00009. They were also more aware of odors in their surroundings, χ^2^(1, *n* = 2107) = 51.40, *p* = 0.000000000001. In contrast, those with autism/ASD were more likely to be suspected of being deaf, χ^2^(1, *n* = 2122) = 36.36, *p* = 0.000000002. See Table 3.

### 3.4. Cognition

Consistent with the research literature, there were significant differences between the two groups with respect to numerous cognition-related issues. Individuals with Asperger syndrome were more likely to be thought of as highly intelligent during their first year of life, χ^2^(2, *n* = 2121) = 51.52, *p* = 0.000000000006, and had reasonable vocabulary skills at a young age, χ^2^(3, *n* = 2098) = 186.06, *p* = 0.0000000000001. In contrast, several questions indicated that those with autism/ASD were less likely to be attentive to their physical and social surroundings including seems “in a shell” “lost in thought,” or difficult to reach, χ^2^(2, *n* = 2115) = 97.66, *p* = 0.0000000000001, looks or walks through people as though they were not there, χ^2^(2, *n* = 2119) = 28.70, *p* = 0.0000006, and inability to direct attention to objects located in a distance or out of a window, χ^2^(2, *n* = 2102) = 74.17, *p* = 0.000000000001. See Table 4.

### 3.5. Medically Related Comorbidities

There were similar rates of seizures, χ^2^(1, *n* = 1796) = 3.28, *p* = 0.07, constipation, χ^2^(1, *n* = 2139) = 0.05, *p* = 0.83, diarrhea, χ^2^(1, *n* = 2139) = 0.96, *p* = 0.33, sleep issues, χ^2^(2, *n* = 2090) = 4.29, *p* = 0.12, and eating challenges, χ^2^(1, *n* = 2039) = 0.27, *p* = 0.60, among individuals in the two groups. None of these findings were statistically significant. See Table 5.

Table 6 summarizes the similarities and differences between those diagnosed with Asperger syndrome and those diagnosed with autism/ASD.

## 4. Discussion

Numerous similarities and differences were identified between those diagnosed with Asperger syndrome and those with autism/ASD, all of which were consistent with both the previous version of the DSM (DSM-IV-TR) and the current version (DSM-V). 

In regard to their similarities, there were no differences with respect to sex ratio, a finding that is in agreement with the 4:1 ratio reported by the Autism and Developmental Disabilities Monitoring Network funded by the Centers for Disease Control and Prevention [33]. Both groups also shared early observable physical signs of autism, such as rhythmic and rocking behavior as well as painful sensitivity to certain sounds. In addition, both groups had a tendency to spin and line up objects and exhibit echolalic speech.

Over the past two decades, researchers have documented a number of medical comorbidities associated with Asperger syndrome and autism/ASD [13,34]. A comparison in this survey indicated no differences between the two groups. This included seizure activity, GI disturbances, hours of daily sleep, and challenges in eating. Regarding GI issues, nearly half suffered from constipation and nearly one-third suffered from diarrhea, with a number suffering from both.

There were also differences between individuals diagnosed with Asperger syndrome and those diagnosed with autism/ASD. This included having first-degree relatives on the autism spectrum in addition to challenges in motor activity, sensory processing, and cognition.

Individuals with Asperger syndrome were more likely to have a parent and/or sibling on the autism spectrum. This is consistent with the genetic literature, in which more than 100 genes have been associated with autism [35]. A possible reason for the difference is that the diagnostic category of Asperger syndrome may reflect a rather homogeneous population, whereas autism/ASD consists of a rather wide distribution of individuals who likely belong to numerous, yet-to-be identified subtypes.

There was relatively little difference between the two groups in regard to having a relative with schizophrenia, and a relatively moderate, but not statistically significant, difference in regard to having a relative with depression.

Individuals diagnosed with Asperger syndrome were more likely to be described as having poor or below average motor coordination. In comparison, those with autism/ASD were more likely to be described as “unusually graceful” [24]. 

Individuals with autism/ASD were more likely to hold their hands in “strange” postures than those with Asperger syndrome. This behavior has sometimes been considered a form of repetitive or stereotypic behavior. However, recent clinical reports suggest that posturing behavior may be a sign of gastrointestinal distress in a number of cases [36,37]. 

Interestingly, those with Asperger syndrome exhibited more hyper-responsiveness to a wide range of sensory sources than those with autism/ASD. This included auditory, olfactory, tactile, and visual stimuli. In contrast, individuals with autism/ASD were more likely to be under-responsive to auditory and visual stimuli. Although sensory sensitivities have been well-documented in early writings on autism [24] and in autobiographies written by autistic individuals [38,39], the fifth edition of the DSM was the first to formally acknowledge unusual sensory sensitivities in those with ASD. 

Many of those with autism/ASD reacted to painful sounds; however, these individuals were also more likely than individuals diagnosed with Asperger syndrome to be suspected of suffering from a hearing impairment. Historically, suspicion of a child possibly being deaf is commonly reported by parents. This may reflect an impairment in attention rather than a sensory-related insensitivity to sounds [40]. 

The results indicate that individuals with autism/ASD are more cognitively impaired with respect to attention to their surrounding environment. They were more likely to be described as “appears as though ‘in a shell’” “lost in thought,” “look(s) through people,” “walk(s) through people,” and “rarely see(s) things very far out of reach.” In contrast, individuals diagnosed with Asperger syndrome were seen as highly intelligent and had age-appropriate vocabulary at a very young age.

Overall, the findings in this survey are consistent with the core features of classical autism [24,31] as well as those associated with Asperger syndrome [41,42]. Symptoms and traits associated with the former included repetitive behaviors (rocking, spinning, and lining up objects) and language (echolalia) in addition to impaired social relatedness (dislikes being touched, avoids people). Symptoms and traits unique to Asperger syndrome included no apparent cognitive impairment early in life, “hyper” sensory responsiveness, and coordination issues. 

It is important to comment, as is evident in the past and present criteria for autism, that no one feature defines autism. That is, the presence of one specific symptom or behavior does not mean an individual is on the autism spectrum. This is referred to as an ill-defined category in the classification literature [43]. 

Moreover, identifying the high (but not perfect) co-occurrence of two or more traits is a relatively accurate approach to classifying categories [44,45]. Reliance on correlations among two or more traits is likely to increase accuracy in diagnosis. Thus, the finding of several traits that distinguish Asperger syndrome from autism/ASD may be of significant importance diagnostically.

In summary, while this study detected similarities between autism/ASD and Asperger syndrome, it also revealed significant differences. Combined with mounting evidence of biomarkers that differentiate Asperger syndrome from autism/ASD, these findings weigh in favor of reinstating Asperger syndrome as a DSM diagnosis. In addition to allowing for more accurate diagnosis, this would encourage researchers to take an interest in studying individuals who do not meet the “acceptable” diagnostic criteria for autism. For instance, it may be enlightening to study the possible hereditary component of Asperger syndrome, given the relatively high number of first-degree relatives on the spectrum reported for individuals with Asperger syndrome in this study. In addition, it would allow many individuals who do not meet the current restrictive criteria of ASD to qualify for needed therapeutic services.

There were several limitations in this study. Although the ages of those diagnosed with Asperger syndrome were consistent with the time period in which the DSM-IV-TR was approved by the American Psychiatric Association, i.e., 1994 to 2012, some of the ASD individuals in this survey may have received a diagnosis of Asperger syndrome if they had been formally diagnosed during this period. 

Second, those individuals who were older than six years of age were grouped into one response category. A detailed breakdown of all ages would have been very helpful in understanding the developmental progression of symptoms and behaviors over the years for those with Asperger syndrome as well as those with autism/ASD. 

Third, all respondents had to have access to the internet, and some individuals may have been hesitant to share personal information online. In addition, not all of the questions were answered. In such cases, users may have deliberately or accidentally skipped these questions. 

## 5. Conclusions

The findings of this study are consistent with the criteria described in the DSM-IV-TR for autistic disorder and for Asperger syndrome, and in the DSM-V for autism spectrum disorder. While the wide range of overlap between Asperger syndrome and autism/ASD clearly demonstrates the similarities between them, the differences suggest that individuals with Asperger syndrome also exhibit distinguishable characteristics. Overall, these findings are consistent with the concept of Asperger syndrome as a subtype of ASD.

Currently, a diagnosis of ASD based on the DSM-V is based solely on observable behaviors. There are no established biologically based criteria to formalize a diagnosis of ASD. Given the current interest in identifying biomarkers to subtype autism [46,47], it may be possible to find associations between a behavioral profile and one or more biomarkers consistent with the criteria for Asperger syndrome as described in the DSM-IV-TR. Consequently, researchers in this and related areas should continue to assess behaviors associated with Asperger syndrome.

## Figures and Tables

**Table 1:** Genetic Features *.

		Asperger	ASD
a. Sex Bias	Boy	81.7%/*n* = 205	83.9%/*n* = 1576
	Girl	18.3%/*n* = 46	16.1%/*n* = 303
b. First Degree Relatives			
On mother’s side	Yes	18.7%/*n* = 47	9.2%/*n* = 173
	No	81.3%/*n* = 204	90.8%/*n* = 1715
On father’s side	Yes	26.7%/*n* = 67	14.4%/*n* = 271
	No	73.3%/*n* = 184	85.6%/*n* = 1617
c. First and Second Degree Relatives	Schizophrenia	7.6%/*n* = 19	9.1%/*n* = 172
	Depressive	42.6%/*n* = 107	31.6%/*n* = 597

* The study was done on 2139 cases (251 children with a diagnosis of Asperger Syndrome and 1888 with Autism Spectrum Disorders), so distributed: <2 y (2.4%), 3–4 y (7.8%), 4–5 y (7.6%), 5–6 y (10.7%) and >6 y 71.5%.

**Table 2:** Early Signs (age ≤2 years).

		Asperger	ASD
a. Spins jar lids, coins, coasters, etc.	Yes, and for long periods	27.7%/*n* = 69	32.9%/*n* = 609
	Very seldom or never	72.3%/*n* = 180	67.1%/*n* = 1240
b. Lines up things and insists he is not disturbed	Yes	65.6%/*n* = 154	59.0%/*n* = 1022
	No	34.4%/*n* = 81	41.0%/*n* = 709
c. Engages in rocking or rhythmic activity for long periods of time	Yes	38.5%/*n* = 95	44.0%/*n* = 816
	Seldom	21.1%/*n* = 52	20.0%/*n* = 373
	No	40.5%/*n* = 100	37.0%/*n* = 678
d. Rocked in crib as a baby	Yes, quite a lot	11.7%/*n* = 24	8.8%/*n* = 145
	Yes, sometimes	15.5%/*n* = 32	12.9%/*n* = 213
	No or very little	72.8%/*n* = 150	78.3%/*n* = 1295
e. During first 2 years, liked to be held?	Yes	64.3%/*n* = 151	68.9%/*n* = 1240
	Limp and passive	4.0%/*n* = 9	6.0%/*n* = 107
	Only when and how he preferred	25.1%/*n* = 59	18.0%/*n* = 325
	Awkward and stiff	7.0%/*n* = 16	7.2%/*n* = 129
f. Avoids people	True/Very True	52.4%/*n* = 129	51.2%/*n* = 958
	False	47.6%/*n* = 117	48.8%/*n* = 913
g. Repeats questions or conversations without variation	True/Very True	42.2%/*n* = 103	37.4%/*n* = 694
	False	57.8%/*n* = 141	62.6%/*n* = 1164
h. Certain sounds seem painful	True/Very True	78.0%/*n* = 192	72.7%/*n* = 1360
	False	22.0%/*n* = 54	27.3%/*n* = 511

**Table 3:** Commonly Reported Descriptors.

		Asperger	ASD
1. Age 3–5: How well physically coordinated?	Unusually graceful	4.4%/*n* = 11	13.3%/*n* = 247
	Above average	16.9%/*n* = 42	31.2%/*n* = 578
	Below average or poor	78.7%/*n* = 196	55.5%/*n* = 1029
2. Age 2–4: Holds hands in strange postures	Yes	41.9%/*n* = 104	57.2%/*n* = 1069
	No	58.1%/*n* = 144	42.8%/*n* = 799
1. In the first year, reacted to bright lights and colors, unusual sounds, etc.	Unusually strong	34.8%/*n* = 87	18.6%/*n* = 348
	Unusually unresponsive	5.6%/*n* = 14	11.5%/*n* = 215
	Average or don’t know	59.6%/*n* = 149	70.0%/*n* = 1313
2. Dislikes being touched or held	True/Very True	47.6%/*n* = 117	30.9%/*n* = 576
	False	52.4%/*n* = 129	69.1%/*n* = 1287
3. Age 3–5: Readily accepts new articles of clothing	Usually resists new clothes	47.6%/*n* = 119	34.9%/*n* = 651
	Doesn’t seem to mind or enjoys them	52.4%/*n* = 131	65.1%/*n* = 1216
4. Intensely aware of odors	True/Very True	63.2%/*n* = 156	39.2%/*n* = 729
	False	36.8%/*n* = 91	60.8%/*n* = 1131
5. Child suspected as very nearly deaf	Yes	25.2%/*n* = 63	45.3%/*n* = 848
	No	74.8%/*n* = 187	54.7%/*n* = 1024

**Table 4:** Cognition.

		Asperger	ASD
a. Age 3–4: Seems “in a shell” “lost in thought” or difficult to reach	Yes	17.7%/*n* = 44	47.5%/*n* = 886
	Occasionally	47.6%/*n* = 118	37.4%/*n* = 698
	No	34.7%/*n* = 86	15.2%/*n* = 283
b. Age 2–4: Looks or walks through people as though they were not there	Yes, often	27.4%/*n* = 68	42.8%/*n* = 800
	Yes, I think so	33.1%/*n* = 82	31.8%/*n* = 595
	No	39.5%/*n* = 98	25.4%/*n* = 476
c. Age 3–5: Attention can be directed to an object some distance away or out a window	Yes	69.5%/*n* = 171	41.1%/*n* = 763
	Rarely sees things far out of reach	30.1%/*n* = 74	53.4%/*n* = 992
	Examines things with fingers & mouth only	0.004%/*n* = 1	5.4%/*n* = 101
d. During first year, seemed unusually intelligent	Suspected high	50.2%/*n* = 124	28.9%/*n* = 541
	Suspected average	47.0%/*n* = 116	60.9%/*n* = 1142
	Suspected lower than average	2.8%/*n* = 7	10.2%/*n* = 191
e. Age 3–5: Vocabulary is out of proportion to his ability to communicate	Points to many named objects, but doesn’t speak or “communicate”	11.2%/*n* = 28	21.6%/*n* = 400
	Correctly names many objects, but does not “communicate”	24.9%/*n* = 62	40.7%/*n* = 752
	Ability to “communicate” is pretty good	63.5%/*n* = 158	23.3%/*n* = 430
	Doesn’t use or understand words	0.004%/*n* = 1	14.4%/*n* = 267

**Table 5:** Medically Related Comorbidities.

		Asperger	ASD
a. Has had seizures	Yes	15.5%/*n* = 35	20.6%/*n* = 324
	No	94.5%/*n* = 191	79.4%/*n* = 1246
b. Constipation	Yes	48.2%/*n* = 121	48.9%/*n* = 924
	No	51.8%/*n* = 130	51.1%/*n* = 964
c. Diarrhea	Yes	30.3%/*n* = 76	33.4%/*n* = 630
	No	69.7%/*n* = 175	66.6%/*n* = 1258
d. Average amount of sleep in a 24-h period	About 8 h	38.4%/*n* = 94	40.9%/*n* = 755
	Less than 8 h	24.1%/*n* = 59	18.5%/*n* = 342
	More than 8 h	37.6%/*n* = 92	40.5%/*n* = 748
e. Eating oddities, such as eating 1 or 2 foods, eating only hot or cold food	Yes	51.9%/*n* = 124	53.7%/*n* = 966
	No	48.1%/*n* = 115	46.3%/*n* = 834

**Table 6:** Similarities and Differences between Asperger Syndrome and Autism/ASD.

		Differences	
	Similarities	Asperger	ASD
Genetics	Male/female ratio	First degree blood relatives	
	Relatives with schizophrenia or depressive		
Early Signs	Spinning and lining up objects		
	Rhythmic or rocking behavior		
	Issues with being held or touched		
	Avoids people		
	Repeats questions/conversations		
	Certain sounds perceived as painful		
Medical Comorbidities	Seizures		
	Constipation and diarrhea		
	Hours of daily sleep		
	Eating oddities		
Commonly Reported Descriptors		Physical coordination issues	Hand-posturing
		Hyper-sensitive to lights, smells, sounds, and touch	Hypo-responsive to lights and sounds
Cognition		Suspected high intelligence	Inattentive to physical and social surroundings
		Communicates with others	

## Data Availability

The data supporting reported results can be found at doi:10.17632/xzbw2gvc4w.1.

## References

[1] American Psychiatric Association (1994). Diagnostic and Statistical Manual of Mental Disorders.

[2] American Psychiatric Association (2000). Diagnostic and Statistical Manual of Mental Disorders.

[3] American Psychiatric Association (2013). Diagnostic and Statistical Manual of Mental Disorders.

[4] Lord C., Jones R.M. (2012). Re-thinking the classification of autism spectrum disorders. J. Child. Psychol. Psychiatry.

[5] Harmon A. (2021). A Specialists’ Debate on Autism Has Many Worried Observers. NYT.

[6] McPartland J.C., Reichow B., Volkmar F.R. (2012). Sensitivity and specificity of proposed DSM-5 diagnostic criteria for autism spectrum disorder. J. Am. Acad. Child Adolesc. Psychiatry.

[7] Spillers J.L.H., Sensui L.M., Linton K.F. (2014). Concerns about identity and services among people with autism and Asperger’s regarding DSM-5 changes. J. Soc. Work. Disabil. Rehabil..

[8] Edelson S.M. (2011). Redefining autism: Confusion or clarity, what outcome?. Autism Res. Rev. Inter..

[9] Bennett M., Goodall E. (2016). A meta-analysis of DSM-5 autism diagnosis in relation to DSM-IV and DSM-IV-TR. Rev. J. Autism Dev. Disord..

[10] Smith I.C., Reichow B., Volkmar F.R. (2017). The effects of DSM-5 criteria on number of individuals diagnosed with autism spectrum disorder: A systematic review. J. Autism Dev. Disord..

[11] Young R.L., Rodi M.L. (2014). Redefining autism spectrum disorder using DSM-5: The implications of the proposed DSM-5 criteria for autism spectrum disorders. J. Autism Dev. Disord..

[12] Qi S., Morris R., Turner J.A., Fu Z., Jiang R., Deramus T.P., Zhi D., Calhoun V.D., Sui J. (2020). Common and unique multimodal covarying patterns in autism spectrum disorder subtypes. Mol. Autism.

[13] Vargason T., Frye R.E., McGuinness D.L., Hahn J. (2019). Clustering of co-occurring conditions in autism spectrum disoder during early childhood: A retrospective analysis of medical claims data. Autism Res..

[14] Faridi F., Khosrowabadi R. (2017). Behavioral, cognitive and neural markers of Asperger syndrome. Basic Clin. Neurosci..

[15] González-Peñas J., Costas J.C., García-Alcón A., Penzol M.J., Rodríguez J., Rodríguez-Fontenla C., Alonso-González A., Fernández-Prieto M., Carracedo Á., Arango C. (2020). Psychiatric comorbidities in Asperger syndrome are related with polygenic overlap and differ from other autism subtypes. Transl. Psychiatry.

[16] Warrier V., Baron-Cohen S., Chakrabarti B. (2013). Genetic variation in GABRB3 is associated with Asperger syndrome and multiple endophenotypes relevant to autism. Mol. Autism.

[17] Shamay-Tsoory S.G., Lester H., Chisin R., Israel O., Bar-Shalom R., Peretz A., Tomer R., Tsitrinbaum Z., Aharon-Peretz J. (2005). The neural correlates of understanding the other’s distress: A positron emission tomography investigation of accurate empathy. Neuroimage.

[18] Kamand M., Ilieva M., Forsberg S.L., Thomassen M., Meyer M., Svenningsen Å.F., Michel T.M. (2020). Derivation of induced pluripotent stem cells (SDUKIi003-A) from a 20-year-old male patient diagnosed with Asperger syndrome. Stem Cell Res..

[19] McAlonan G.M., Daly E., Kumari V., Critchley H.D., Van Amelsvoort T., Suckling J., Simmons A., Sigmundsson T., Greenwood K., Russell A. (2002). Brain anatomy and sensorimotor gating in Asperger’s syndrome. Brain.

[20] Murphy C.M., Deeley Q., Daly E., Ecker C., O’Brien F., Hallahan B., Loth E., Toal F., Reed S., Hales S. (2012). Anatomy and aging of the amygdala and hippocampus in autism spectrum disorder: An in vivo magnetic resonance imaging study of Asperger syndrome. Autism Res..

[21] Howlin P. (2003). Outcome in high-functioning adults with autism with and without early language delays: Implications for the differentiation between autism and Asperger syndrome. J. Autism Dev. Disord..

[22] Mazzone L., Ruta L., Reale L. (2012). Psychiatric comorbidities in Asperger syndrome and high functioning autism: Diagnostic challenges. Ann. Gen. Psychiatry.

[23] de Giambattista C., Ventura P., Trerotoli P., Margari M., Palumbi R., Margari L. (2019). Subypting the autism spectrum disoder: Comparison of children with high functioning autism and Asperger syndrome. J. Autism Dev. Disord..

[24] Rimland B. (1964). Infantile Autism: The Syndrome and its Implications for a Neural Theory of Behavior.

[25] Cohen D.J., Caparulo B.K., Gold J.R., Waldo M.C., Shaywitz B.A., Ruttenberg B.A., Rimland B. (1978). Agreement in diagnosis: Clinical assessment and behavior rating scales for pervasively disturbed children. J. Am. Acad. Child Psychiatry.

[26] Leddet I., Larmande C., Barthelemy F., Sauvage D., Lelord G. (1986). Comparison of clinical diagnoses and Rimland E2 scores in severely disturbed children. J. Autism Dev. Disord..

[27] Edelson S.M. (2021). Comparison of autistic individuals who engage in self-injurious behavior, aggression, and both behaviors. Pediatr. Rep..

[28] Edelson S.M., Edelson S.M., Johnson J.B. (2021). Conclusion. Understanding and Treating Anxiety in Autism.

[29] Konstantareas M.M., Homatidis S. (1992). Mothers’ and fathers’ self-report of involvement with autistic, mentally delayed, and normal children. J. Marriage Fam..

[30] Panter-Brick C., Burgess A., Eggerman M., McAllister F., Pruett K., Leckman J.F. (2014). Practitioner review: Engaging fathers—Recommendations for a game change in parenting interventions based on a systematic review of the global evidence. J. Child Psychol. Psychiatry.

[31] Kanner L. (1943). Autistic disturbances of affective contact. Nerv. Child..

[32] Doshi-Velez F., Ge Y., Kohane I. (2014). Comorbidity clusters in autism spectrum disorders: An electronic health record time-series analysis. Pediatrics.

[33] Baio J., Wiggins L., Christensen D.L., Maenner M.J., Daniels J., Warren Z., Kurzius-Spencer M., Zahorodny W., Rosenberg C.R., White T. (2018). Prevalence of autism spectrum disorder among children aged 8 years—Autism and developmental disabilities monitoring network, 11 sites, United States, 2014. MMWR Surveill. Summ..

[34] Muskens J.B., Velders F.P., Staal W.G. (2017). Medical comorbidities in children and adolescents with autism spectrum disorders and attention deficit hyperactivity disorders: A systematic review. Eur. Child Adolesc. Psychiatry.

[35] Satterstrom F.K., Kosmicki J.A., Wang J., Breen M.S., De Rubeis S., An J.Y., Peng M., Collins R., Grove J., Klei L. (2020). Large-scale exome sequencing study implicates both developmental and functional changes in the neurobiology of autism. Cell.

[36] Buie T., Campbell D.B., Fuchs G.J., Furuta G.T., Levy J., VandeWater J., Whitaker A.H., Atkins D., Bauman M.L., Beaudet A.L. (2010). Evaluation, diagnosis, and treatment of gastrointestinal disorders in individuals with ASDs: A consensus report. Pediatrics.

[37] Wasilewska J., Klukowski M. (2015). Gastrointestinal symptoms and autism spectrum disorder: Links and risks—a possible new overlap syndrome. Pediatric Health Med. Ther..

[38] Grandin T., Scariano M.M. (1986). Emergence: Labeled Autistic.

[39] Williams D. (1992). Nobody Nowhere.

[40] Koegel R.L., Schreibman L. (1976). Identification of consistent responding to auditory stimuli by a functionally “deaf” autistic child. J. Autism Child. Schizophr..

[41] Asperger H. (1944). Die “Autistischen Psychopathen” im Kindesalter. Arch. Psychiatr. Nervenkrankh..

[42] Wing L. (1981). Asperger’s syndrome: A clinical account. Psychol. Med..

[43] Smith E.E., Medin D.L. (1981). Categories and Concepts.

[44] Medin D.L., Altom M.W., Edelson S.M., Freko D. (1982). Correlated symptoms and simulated medical classification. J. Exp. Psychol. Learn. Mem. Cogn..

[45] Medin D.L., Edelson S.M. (1988). Problem structure and the use of base rate information from experience. J. Exp. Psychol. Gen..

[46] Frye R.E., Vassall S., Kaur G., Lewis C., Karim M., Rossignol D. (2019). Emerging biomarkers in autism spectrum disorder: A systematic review. Ann. Transl. Med..

[47] Geurts H.M., van Rentergem J.A.A., Radhoe T., Torenvliet C., Van der Putten W.J., Groenman A.P. (2021). Ageing and heterogeneity regarding autism spectrum conditions: A protocol paper on an accelerated longitudinal study. BMJ Open.

